# Propranolol and breast cancer—a work in progress

**DOI:** 10.3332/ecancer.2018.ed82

**Published:** 2018-06-18

**Authors:** Pan Pantziarka, Brad A Bryan, Sergio Crispino, Erin B Dickerson

**Affiliations:** 1 Anticancer Fund, Brussels, 1853 Strombeek-Bever, Belgium; 2 The George Pantziarka TP53 Trust, London, UK; 3 Department of Biomedical Sciences, Texas Tech University Health Sciences Center, El Paso, Texas, USA; 4 Department of Veterinary Clinical Sciences, University of Minnesota, Saint Paul, Minnesota, USA; 5 Masonic Cancer Center, University of Minnesota, Minneapolis, Minnesota, USA

**Keywords:** drug repurposing, propranolol, beta blocker, breast cancer, perioperative therapies

## Abstract

The non-selective beta-blocker propranolol is a leading candidate for repurposing as a novel anti-cancer agent. Emerging evidence, including human data, suggests that there are multiple mechanisms of action particularly relevant to breast cancer. This editorial reviews a number of recent studies that show it has anti-metastatic activity that warrants clinical investigation, including investigation as a potential perioperative therapy in breast cancer.

## Introduction

The non-selective beta-blocker propranolol is a drug repurposing success story. Originally developed by James W. Black at ICI in the early 1960s, the drug is now routinely used for a wide range of medical indications – from hypertension to infantile hemangioma to thyrotoxicosis and more. It is also emerging as a significant repurposing candidate in oncology. As with many drugs developed in a more empirical era of drug development, propranolol is relatively non-selective in its targets – in this case the beta adrenergic receptors. However, this non-selectivity is a virtue rather than a defect – in an age of targeted drugs, propranolol, like many other venerable and widely used repurposing candidates, can be viewed as a multi-targeted agent. This translates into multiple relevant mechanisms of action – anti-proliferative, anti-angiogenic, anti-lymphangiogenic, pro-apoptotic, immunomodulating – with evidence from a wide range of data sources and cancer types [1]. Significant work by Ben-Eliyahu [2–4], Sood [5–7], Sloan [8, 9] and their colleagues and collaborators have extensively explored the connections between beta-adrenergic signalling, surgical stress and cancer proliferation and metastasis in a wide range of malignancies.

These mechanisms of action suggest that propranolol may be of use in different cancer treatment settings – neo-adjuvant, perioperative and adjuvant. In particular, this constellation of mechanisms suggests that propranolol might be most significant as an anti-metastatic agent in many cancers. Emerging evidence for these anti-metastatic effects is particularly interesting in breast cancer – where reducing metastatic spread will effectively yield an increase in lives saved.

## Retrospective data

There is existing retrospective data suggestive of a protective effect of propranolol in breast cancer. In an analysis of women diagnosed with Stage I – IV breast cancer in Ireland between 2001 and 2006, Barron and colleagues found that compared to matched controls women taking propranolol had a reduced incidence of locally invasive (T4) or metastatic (N2/N3/M1) tumours at the time of diagnosis [10]. The reduction in metastatic spread was especially notable (OR, 0.20; 95% CI, 0.04 to 0.88), and there was a corresponding reduction in the risk of cancer-specific mortality (HR, 0.19; 95% CI, 0.06 to 0.60). In contrast, there were no differences between matched controls and women taking the selective beta-blocker atenolol. A later meta-analysis by Childers *et al* did not differentiate between selective and non-selective beta blockers but also reported a significant reduction in breast cancer mortality (HR, 0.50; 95% CI, 0.32-0.80) [11]. However, a pooled European analysis by Cardwell *et al* found no evidence for a protective effect of propranolol or beta-blockers in general – although the study did not assess outcomes by stage or primary vs metastatic disease, which may be important [12].

## Recent studies

Evidence for anti-metastatic effects also comes from a range of investigators pursuing several lines of both preclinical and clinical research. A number of recent publications, (in the period since 2016), focus on breast cancer in particular.

Rico *et al* showed that propranolol reduced cell viability and migration in a panel of breast cancer cell lines, and that the effect was increased when combined with metformin, another high-profile repurposing candidate [13]. Furthermore, the combination reduced tumour growth in two immunocompetent models of triple-negative breast cancer, thereby improving survival. Treatment also reduced metastatic growth, with evidence that propranolol reduced colonisation in the lungs.

A team at Texas Tech University Health Sciences Center retrospectively assessed the impact of selective and non-selective beta-blockers on tumour proliferation (Ki67) [14]. Results showed that non-selective beta blockade reduced tumour proliferation by 66% in early stage breast cancer. Cell line data showed that propranolol dose dependently reduced tumour cell viability. Data from a Stage I patient prospectively treated with propranolol for three weeks showed that Ki67 staining was reduced by 23%.

These results were in line with data from a small (n=38) Phase II randomised placebo-controlled trial (NCT00502684) of perioperative propranolol in combination with etodolac, another repurposing candidate, in women with early stage breast cancer [15]. Women in the treatment group were treated with the drug combination for 11 days, starting five days before surgical resection. Results showed that the treatment decreased epithelial-to-mesenchymal transition, reduced activity of pro-metastatic/pro-inflammatory transcription factors and decreased tumour-infiltrating monocytes while increasing tumour-infiltrating B cells.

Psychological stress following breast cancer diagnosis, and particularly around the time of surgery may be a factor in some of these results. This was nicely illustrated by Budiu *et al*, who showed in a mouse model of breast cancer that stress (induced by social isolation of mice) decreased survival through immune-mediated mechanisms [16]. This latest finding is firmly in line with previous work, which has shown beta-adrenergic signalling can induce a significant pro-metastatic environment in primary breast cancer [7]. More recent work has shown that chronic stress and increased beta-adrenergic signalling leads to the creation of a ‘pre-metastatic’ niche in the lungs, thereby facilitating the colonisation of the lungs by circulating breast cancer cells [17]. This niche formation was inhibited by propranolol.

It is also known that propranolol can impact the immunosuppression associated with elevated stress signalling. Zhou *et al* reported on the effect of propranolol on immune function in women undergoing breast cancer surgery [18]. Women (n=101) were randomised to propranolol, parecoxib, propranolol + parecoxib, or control – blood was collected pre-operatively and at multiple time points to seven days post-op. Where the control group showed elevated numbers of immunosuppressive T-regulatory cells, patients in the propranolol and propranolol + parexcoxib group showed no such increase.

Indeed, the immunological effects have also been explored by Ashrafi and colleagues *in vivo*, who showed that propranolol and vaccination with tumour antigen lysate increased survival of tumour-bearing mice [19]. The addition of propranolol increased IL-2, IL-4, IL-12, IL-17, and IFN-γ cytokines compared to treatment with vaccine alone.

Other recent work by Sood and colleagues has also investigated the relationship between beta adrenergic receptor signalling and the tumour stroma [20]. Using data from ovarian, colon and breast cancers it was shown that stress signalling induced phenotypic changes associated with pro-tumour and pro-metastatic cancer-associated fibroblasts and increased collagen deposition in tumours. These effects were abrogated with propranolol treatment.

## Breast cancer subtypes

In terms of breast cancer subtypes there is some evidence that the effects are independent of hormone receptor status [14]. Some initial work, mainly retrospective, has explored the use of beta-blockers and specific subtypes. Retrospective studies have shown that beta-blocker usage is associated with improved recurrence free survival in women with triple-negative breast cancer (TNBC) [21, 22] and reduced risk of metastasis [22]. In a trial of advanced HER2 negative breast cancer, beta-blockers were associated with improved progression free survival (PFS), particularly for the subgroup of TNBC patients [23]. There is also some evidence to suggest that propranolol may revert resistance to trastuzumab in HER2 positive breast cancer [24]. While there are many questions yet to be explored, it is clear that propranolol may have therapeutic value generally in breast cancer.

## Impact on metastatic sites

Metastatic spread to the bones is of particular concern in breast cancer, and here too evidence exists that propranolol has some impact. Campbell *et al* have shown that activation of the sympathetic nervous system promotes colonisation of breast cancer cells to bone via neurohormonal effects on the bone marrow stroma [25, 26]. They showed *in vivo* that propranolol abrogated this pro-metastatic process in tumour-bearing mice.

Propranolol was also shown to have an effect on metastasis to the brain – the other major site of interest in breast cancer. Choy *et al* assessed retrospective data that showed that for stage II breast cancer patients beta-blocker usage was associated with a significantly reduced risk of post-operative recurrence or distant metastasis (HR 0.51; 95% CI: 0.23-0.97; P=0.041) [27]. In vitro analysis showed that primary and brain metastatic triple negative cancer cells showed high expression of beta2-adrenergic receptors, and that these promoted metastasis to the brain. In vivo experiments showed that mice injected with cells pre-treated with propranolol had significantly fewer brain metastases than control mice.

## Other beta-blockers

Of course there are many other beta blockers in widespread clinical use, both non-selective (for example carvedilol) and selective (examples include β1-selective atenolol). While the majority of preclinical studies have focused on propranolol there is also some evidence for anticancer effects for carvedilol [28, 29], atenolol [30] and others. These drugs vary by the degree of beta adrenergic receptor selectivity and range of off-target effects, and it remains to be seen to what extent these impact the anticancer potential of these drugs. Certainly there is evidence that different cancer types exhibit differences in beta adrenergic receptor expression [31], suggesting that drugs which have an inhibitory activity more closely aligned to tumour characteristics may show greater therapeutic effect. It is an interesting question to explore to what extent breast cancer subtypes differ in their beta adrenergic receptor expression.

## Conclusion

The recent studies outlined in this paper add to the weight of evidence to support the use of propranolol as an anti-metastatic agent in breast cancer. It may be particularly effective in the neo-adjuvant period or as a perioperative therapy [32]. However, many of propranolol’s putative anticancer mechanisms of action are not tissue specific. There remains a clear potential for propranolol to be useful in a range of other cancers, including angiosarcoma [33–35], melanoma [36], and retinal haemangioblastomas in von Hippel-Lindau disease [37]. Currently there are over 20 active clinical trials in different cancers, settings and countries – showing that for this drug at least, despite the lack of financial incentives [38], the potential for repurposing is being actively pursued by the oncology community.

## Author contributions

Primary author: Pan Pantziarka. Contributing authors: Brad A Bryan, Sergio Crispino and Erin B Dickerson.

All authors read and approved the final manuscript.

## Competing interests

The authors declare that they have no competing interests. No funding was received for this paper.
